# Rabies control in India: a need to close the gap between research and policy

**DOI:** 10.2471/BLT.14.140723

**Published:** 2014-12-18

**Authors:** Syed Shahid Abbas, Manish Kakkar

**Affiliations:** a Institute of Development Studies, University of Sussex, Brighton, England.; b Public Health Foundation of India, ISID Campus, 4 Vasant Kunj Institutional Area, New Delhi, 110070, India.

Rabies – a viral zoonosis– is recognized as a priority disease for global and national level control measures.^1^^–^^3^ Key interventions for rabies control include vaccination for high-risk individuals, surveillance of human cases, post-exposure prophylaxis following animal bites, vaccination and/or culling of the canine population and other animal reservoirs. Despite the known effectiveness of these interventions,^4^ many national policy-makers across Africa and Asia hesitate to introduce them.^5^^,^^6^ We argue that this hesitation may be caused by advocacy for technical solutions that do not account for local requirements of policy-makers and implementers.

There has been a considerable amount of research on rabies in India, but key questions remain unaddressed.^6^ For example, while policy-makers need evidence for social, political and economic outcomes of control programmes, most rabies research is done in the basic sciences.^6^ We aim to illustrate the research–policy disconnect in rabies control and describe how this disconnect contributes to the lack of effective rabies-control policies in India.

Over the past decade, intradermal administration of rabies vaccine for post-exposure prophylaxis has heralded a major change in rabies-control measures. The World Health Organization recommends this route when vaccines are in short supply, citing the 60–80% reduction in direct costs and vaccine consumption, compared with standard intramuscular injection.^4^^,^^7^ While intradermal administration makes efficient use of limited vaccine supplies, the costs saved by using this strategy at a programme level have not been fully ascertained.

We conducted a cost analysis of rabies-control programmes in India and showed that the choice of vaccination route contributed the least to government cost‒savings compared to other drivers of programme costs, such as procurement costs and wastage rates of different vaccine formulations and the incidence of dog bites.^8^ Thus, cost-savings from a centralized procurement and supply-chain management system might be outweighed by the costs of uniform adoption of intradermal vaccination in an Indian setting. Through discussion with the programme managers, we found that they favoured the intradermal route in urban hospitals and the intramuscular route in peripheral health facilities. Peripheral facilities, which have fewer patients than urban hospitals, treat one to two dog bites per week, hence, the multi-dose vaccine vials are discarded after use in one patient. Therefore, the intradermal use policy does not always lead to vaccine savings but could increase the transaction costs of the vaccination due to the increased need for training and supervision of vaccinators.^8^^,^^9^

Canine vaccination is the suggested strategy of choice towards elimination of rabies.^4^^,^^10^ However, we have found little documentation of the expectations of local communities or rabies-control programme managers as to how this strategy would work.^5^^,^^6^ The cost of a canine vaccination programme is three to 10 times higher than the cost of human prophylaxis.^8^ Modelling of pilot canine vaccination interventions in India showed that a coverage of 70% would have to be sustained over two decades in order for the intervention to be effective.^11^ Even at a cost of 0.13 US dollars (US$) per vaccinated dog, the total cost per year for a national programme of canine vaccination would be US$ 23 million – 27% of the total budget of the State Department of Animal Husbandry in 2012. In addition, unless accompanied with dog population control, this strategy is not likely to reduce the number of dog bites or the costs of post exposure prophylaxis.

In India, eliminating a single-host disease such as polio has been logistically challenging and vaccination coverage is still less than 60% in many large states.^12^ Eliminating rabies by vaccinating the entire canine population is understandably a low priority for most decision-makers in the country. 

We do not have good estimates of transmission of rabies from dogs to humans and from dogs to cattle. In 2003, it was estimated that 14 000–17 000 human deaths were caused by rabies in India.^13^ No nationally-representative epidemiological data have been collected since then. Similarly, official statistics report around 300 rabies-related cattle deaths annually for the entire country.^14^ The lack of more reliable information makes it difficult to plan and evaluate the success of any rabies-control measures.

What are the rabies-control solutions for India? Researchers and policy-makers need to jointly promote evidence-based policy-making. The landscape of rabies control is complex; with actors from multiple sectors – including animal welfare, public health, veterinary medicine and civil administration – with different perspectives and expectations of a rabies control programme. In such an environment simple intervention strategies are not likely to meet the needs of all stakeholders. Instead, an approach that recognizes – while seeking to improve – the interdependence of human, animal and environmental health, may bring multiple perspectives together. More epidemiological studies and implementation research should be conducted with a wider set of actors. For us, the recognition of the complexity of rabies control is the first step to developing more effective, acceptable and sustainable policy solutions.
